# Age-Specific Seroprevalence of Dengue IgG Antibodies Among Children, Adolescents, and Young Adults in North India: A Cross-Sectional Study

**DOI:** 10.7759/cureus.104192

**Published:** 2026-02-24

**Authors:** Rajesh Kapoor, Sanjay Verma, Amit Rawat, Vanita Suri, Vikas Suri, Priyanka Khambra

**Affiliations:** 1 Advanced Pediatric Centre, Postgraduate Institute of Medical Education and Research, Chandigarh, IND; 2 Department of Pathology, Postgraduate Institute of Medical Education and Research, Chandigarh, IND; 3 Department of Obstetrics and Gynecology, Postgraduate Institute of Medical Education and Research, Chandigarh, IND; 4 Department of Internal Medicine, Postgraduate Institute of Medical Education and Research, Chandigarh, IND; 5 Department of Obstetrics and Gynecology, Maharaja Agrasen Medical College, Agroha, IND

**Keywords:** adolescents, dengue seroprevalence, igg antibodies, north india, vector-borne diseases

## Abstract

Background

Dengue is a major mosquito-borne viral infection in India, with substantial underestimation of the true infection burden due to asymptomatic and subclinical cases. Seroprevalence studies provide a more reliable estimate of cumulative exposure and population-level immunity. Limited data are available comparing age-specific seroprevalence across late childhood, adolescence, and early adulthood within the same population. This study aimed to determine dengue IgG seroprevalence among children, adolescents, and young adults in a tertiary care setting in North India and to assess its association with selected sociodemographic factors.

Methods

A cross-sectional study with prospective enrollment of participants was conducted from March 2019 to February 2022 after institutional ethics approval. A total of 240 apparently healthy participants aged nine to 30 years were enrolled consecutively and stratified into children (nine to 12 years; n=75), adolescents (15 to 18 years; n=75), and adults (25 to 30 years; n=90). Demographic data were collected using a pre-structured proforma. Serum IgG antibodies against dengue virus (DENV-1 to DENV-4) were detected using the Panbio Dengue IgG indirect enzyme-linked immunosorbent assay (ELISA) (Abbott Panbio Diagnostics, Brisbane, Australia). Seropositivity was defined as Panbio Units (PU) >11. Statistical analysis was performed using SPSS version 25.0 (IBM Corp., Armonk, NY, USA). Chi-square test and one-way ANOVA were applied where appropriate, with p<0.05 considered significant.

Results

The mean age of participants was 18.72 ± 6.93 years. Of the 240 individuals enrolled, 129 (53.8%) were male and 111 (46.3%) were female; 127 (52.9%) resided in rural areas and 113 (47.1%) in urban areas. Overall dengue IgG seropositivity was 51.2% (123/240; 95% CI: 44.9%-57.5%). Age-wise seroprevalence was 37.3% (95% CI: 26.4%-48.2%) among children, 62.7% (95% CI: 51.8%-73.6%) among adolescents, and 53.3% (95% CI: 43.0%-63.6%) among adults. Mean IgG titers differed significantly across age groups (F(2,237)=5.98, p=0.003), with the highest levels observed in adolescents (24.33 ± 19.52 PU), followed by adults (18.44 ± 18.21 PU) and children (13.83 ± 18.26 PU). In bivariate analysis, age group (χ²=9.88, p=0.007) and residence (χ²=8.23, p=0.004) were significantly associated with seropositivity. Urban participants showed higher seroprevalence than rural participants (61.1% vs. 42.5%). Gender (χ²=0.08, p=0.773) and socioeconomic class (χ²=1.10, p=0.578) were not significantly associated. On multivariable analysis, children had lower odds of seropositivity compared to adults (adjusted OR 0.45; 95% CI: 0.23-0.87; p=0.018), while urban residence remained independently associated with higher seropositivity (adjusted OR 2.16; 95% CI: 1.26-3.69; p=0.005).

Conclusion

This study demonstrates a high dengue IgG seroprevalence (51.2%) among individuals aged nine to 30 years in North India, indicating substantial cumulative exposure. Seroprevalence varied significantly across age groups, with adolescents showing the highest exposure and children having significantly lower adjusted odds compared to adults. Urban residence was independently associated with higher seropositivity, suggesting intensified transmission in urban settings. These findings highlight ongoing dengue transmission and emphasize the need for age-stratified surveillance, strengthened vector control, and context-specific vaccination strategies in endemic regions.

## Introduction

Dengue has emerged as one of the fastest-growing mosquito-borne viral infections worldwide, with nearly a 50% increase in global incidence over the past three decades. Currently endemic in more than 100 countries, it represents a major public health concern, particularly in low- and middle-income regions. The World Health Organization (WHO) recognizes dengue as a significant global health threat because of its expanding geographic distribution and recurrent epidemics [1,2]. Infection may occur with any of the four dengue virus serotypes (DENV-1, DENV-2, DENV-3, and DENV-4), which are transmitted through the bite of infected Aedes mosquitoes [3]. Globally, an estimated 96 million clinically apparent cases and approximately 293 million total infections were reported in 2010 [4]. In 2021, the age-standardized global incidence rate reached 752.04 per 100,000 population, reflecting a 47.26% increase since 1992, with Southeast Asia demonstrating the most pronounced rise in incidence [5].

India contributes substantially to the global dengue burden, with more than 100,000 laboratory-confirmed cases reported annually through national surveillance systems [6]. Periodic epidemic peaks are associated with increased hospitalization rates and case fatality. Although dengue was historically concentrated in urban centers, recent surveillance data indicate sustained transmission in peri-urban and rural regions, suggesting geographic expansion of the disease. Rapid urbanization, industrialization, population mobility, and infrastructural development have been implicated in this epidemiological transition [7].

Despite a national surveillance network comprising over 600 sentinel hospitals, reported case numbers likely underestimate the true magnitude of infection. A nationwide serosurvey conducted by Murhekar et al. in 2017 estimated an overall dengue seroprevalence of 48.7% in India, indicating that nearly half of the population had evidence of prior exposure - substantially higher than annual reported case counts [8]. Passive surveillance systems primarily capture symptomatic individuals seeking care in public healthcare facilities, whereas mild, asymptomatic, and subclinical infections frequently remain undetected. Incomplete reporting from the private healthcare sector further limits accurate burden estimation [6].

In this context, seroprevalence studies provide a more reliable assessment of cumulative exposure and population-level immunity than routine case-based surveillance [8]. Although several studies have reported dengue seroprevalence from different regions of India, limited data exist comparing age-specific seroprevalence across late childhood, adolescence, and young adulthood within the same population [9]. Alagarasu et al. demonstrated a progressive increase in immunoglobulin G (IgG) seropositivity from 70.1% in the zero-to-nine-year age group to 85.0% in the 10-to-19-year age group [10]. Similarly, Indu et al. reported increasing seroprevalence with age, rising from 25.7% at nine years to 33.9% at 12 years [11]. These findings highlight the importance of understanding age-related exposure patterns in endemic settings.

Seroprevalence estimates are also relevant for informing vaccination strategies, as currently available dengue vaccines demonstrate greater efficacy in seropositive individuals compared to seronegative individuals. Regularly conducted serosurveys may therefore help identify age groups with higher background immunity and guide optimal vaccination timing. Accordingly, this study aimed to determine age-specific dengue IgG seroprevalence among children, adolescents, and young adults in a tertiary care setting in North India, and to evaluate its association with selected sociodemographic factors, recognizing that IgG seropositivity reflects cumulative exposure rather than confirmed protective immunity.

## Materials and methods

Study design

The present study was a cross-sectional study with prospective enrollment of participants that was conducted in the outpatient departments of Pediatrics and Gynecology at the host institute. The study was approved by the Institutional Ethics Committee (IEC) of Postgraduate Institute of Medical Education and Research (PGIMER), Chandigarh (Letter No. INT/IEC/2019/00226). The study was conducted over a period of three years, from March 2019 to February 2022. Written informed consent was obtained from all adult participants. For participants below 18 years of age, written informed consent was obtained from parents or legal guardians, and assent was obtained from children/adolescents as appropriate.

Participant enrollment

**Figure 1:**
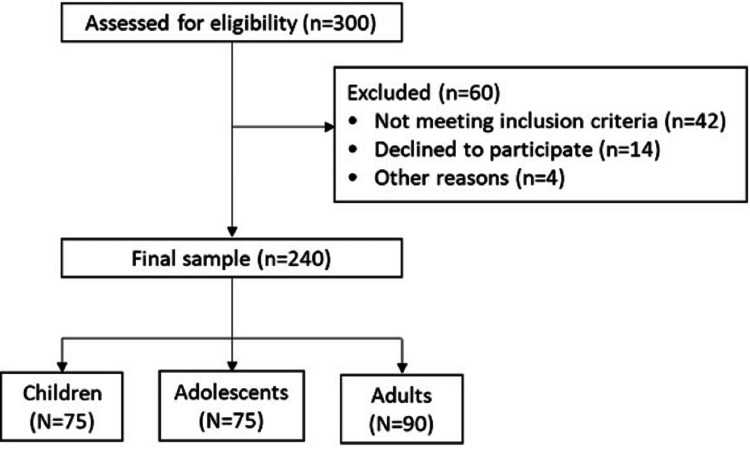
Participant flow diagram.

A total of 300 individuals were screened for eligibility. Sixty individuals were excluded: 42 did not meet the inclusion criteria, 14 declined participation, and four were excluded for other reasons (Figure 1). The final study sample comprised 240 participants. Participants aged nine to 30 years were enrolled consecutively from the outpatient departments during the study period. This age range was selected to evaluate cumulative dengue exposure across late childhood, adolescence, and early adulthood. Participants were subsequently stratified into three epidemiologically relevant categories: nine to 12 years (late childhood), 15 to 18 years (adolescence), and 25 to 30 years (young adulthood). These categories represent distinct behavioral and exposure patterns that may influence dengue transmission dynamics. The final distribution across groups reflects consecutive enrollment rather than predetermined proportional allocation. These predefined age categories were selected to represent distinct epidemiological and behavioral exposure stages. Intermediate age groups were not separately analyzed to maintain clear stratification across developmental phases and due to pragmatic outpatient recruitment patterns.

Sample size calculation

The sample size was calculated using the formula for estimating proportions in cross-sectional studies (n = Z²pq/d²), assuming an expected seroprevalence of 50%, a 95% confidence level (Z = 1.96), and 10% absolute precision. The minimum required sample size was calculated to be 96 participants. A total of 240 participants were enrolled to improve precision and enable subgroup analysis across age categories. An expected seroprevalence of 50% was assumed to yield the maximum sample size in the absence of precise regional estimates for this specific age range. Subgroup analyses were exploratory, and the study was not separately powered for individual age-group comparisons.

Inclusion and exclusion criteria

Apparently healthy individuals aged nine to 30 years who were willing to participate were included in the study. Apparently healthy individuals were defined as participants attending outpatient services for minor non-infectious complaints or routine visits, without symptoms suggestive of acute febrile illness, and without known immunocompromising conditions based on clinical history and medical record review. Participants were excluded if they had a known diagnosis of malignancy or immunodeficiency, were receiving prolonged corticosteroid therapy (more than two weeks), had discontinued corticosteroid therapy within the preceding four weeks, or had received blood transfusion, plasma transfusion, or immunoglobulin therapy within the previous three months, as these conditions could potentially alter antibody responses and affect serological interpretation (Figure 1).

Data collection

Using a pre-structured proforma, relevant demographic and clinical data were collected from participants or from parents/guardians in the case of children and adolescents. Information regarding age, sex, residence, and socioeconomic status was recorded. Socioeconomic status was assessed using the Modified Kuppuswamy Scale (2019 income update applicable at study initiation) [12]. Additionally, the history of dengue, varicella, and hepatitis was documented. The data collection proforma was developed by the investigators for the purpose of this study. The Modified Kuppuswamy Scale is available in the public domain and does not require formal permission for academic use. No copyrighted or proprietary questionnaires were used in this study.

Blood sample collection and storage

Venepuncture was performed to collect 3 mL of venous blood in sterile vacutainers. The blood was allowed to clot at room temperature for 30 minutes, followed by serum separation by centrifugation at 3000 rpm for 10 minutes. Serum samples were stored at −20°C until analysis. Serum samples were analyzed in batches within three months of collection. Samples were not subjected to repeated freeze-thaw cycles.

Estimation of IgG antibodies against dengue

All serum samples were analyzed for IgG antibodies against dengue virus serotypes (DENV-1, DENV-2, DENV-3, and DENV-4). The Panbio Dengue IgG indirect enzyme-linked immunosorbent assay (ELISA) kit (Abbott Panbio Diagnostics, Brisbane, Australia) was used according to the manufacturer’s instructions. Internal quality control was maintained by running manufacturer-provided positive and negative controls with each batch. Panbio Units (PU) were calculated by dividing the specimen absorbance by the kit-specific cutoff value and multiplying the result by 10. As per manufacturer criteria, samples were interpreted as positive when PU >11, negative when PU <9, and equivocal when PU ranged between 9 and 11. No samples fell within the equivocal range. A PU value >11 was considered indicative of seropositivity, reflecting prior dengue virus exposure.

Statistical analysis

Data were entered into Microsoft Excel (Redmond, WA, USA) and analyzed using the Statistical Package for the Social Sciences (SPSS) software version 25.0 (IBM Corp., Armonk, NY, USA). Normality of IgG titers was assessed using the Shapiro-Wilk test. As the distribution showed mild skewness, the results of one-way analysis of variance (ANOVA) were cross-validated using the Kruskal-Wallis test. Continuous variables were expressed as mean ± standard deviation (SD), while categorical variables were presented as frequencies and percentages. The Chi-square test was used to compare seropositivity rates across different age groups and sociodemographic variables. One-way ANOVA was applied to compare mean IgG antibody titers (PU) among the three age groups. A p-value <0.05 was considered statistically significant. Variables with p<0.10 in bivariate analysis and those considered epidemiologically relevant were entered into the multivariable logistic regression model using the enter method.

## Results

A total of 240 participants were enrolled in the study, comprising 75 (31.3%) children aged nine to 12 years, 75 (31.3%) adolescents aged 15 to 18 years, and 90 (37.5%) adults aged 25 to 30 years. The overall mean age was 18.72 ± 6.93 years. The mean age was 10.74 ± 0.98 years in children, 16.88 ± 1.03 years in adolescents, and 26.92 ± 1.74 years in adults. Of the total participants, 129 (53.8%) were male and 111 (46.3%) were female. A majority resided in rural areas (127; 52.9%), while 113 (47.1%) were from urban localities. Regarding socioeconomic status, 86 (35.8%) belonged to the lower class, 153 (63.7%) to the middle class, and one (0.4%) to the upper class. A history of dengue was reported in 15 (6.3%) participants, varicella in 90 (37.5%), and hepatitis in eight (3.3%) participants (Table 1).

**Table 1:** Sociodemographic and clinical determinants of the enrolled participants.

Variable	Subgroup	Children (N=75)	Adolescents (N=75)	Adults (N=90)	Total (N=240)
Age (years)		10.74 ± 0.98	16.88 ± 1.03	26.92 ± 1.74	18.72 ± 6.93
Gender	Male	57 (76%)	33 (44%)	39 (43.3%)	129 (53.8%)
	Female	18 (24%)	42 (56%)	51 (56.7%)	111 (46.3%)
Residence	Rural	40 (53.3%)	33 (44%)	54 (60%)	127 (52.9%)
	Urban	35 (46.7%)	42 (56%)	36 (40%)	113 (47.1%)
Socioeconomic class	Lower	25 (33.3%)	29 (38.7%)	32 (35.6%)	86 (35.8%)
	Middle	50 (66.7%)	46 (61.3%)	57 (63.3%)	153 (63.7%)
	Higher	0	0	1 (1.1%)	1 (0.4%)
History of dengue	Present	0	3 (4%)	12 (13.3%)	15 (6.3%)
History of varicella	Present	14 (18.7%)	32 (42.7%)	44 (48.9%)	90 (37.5%)
History of hepatitis	Present	1 (1.3%)	3 (4%)	4 (4.4%)	8 (3.3%)

**Figure 2:**
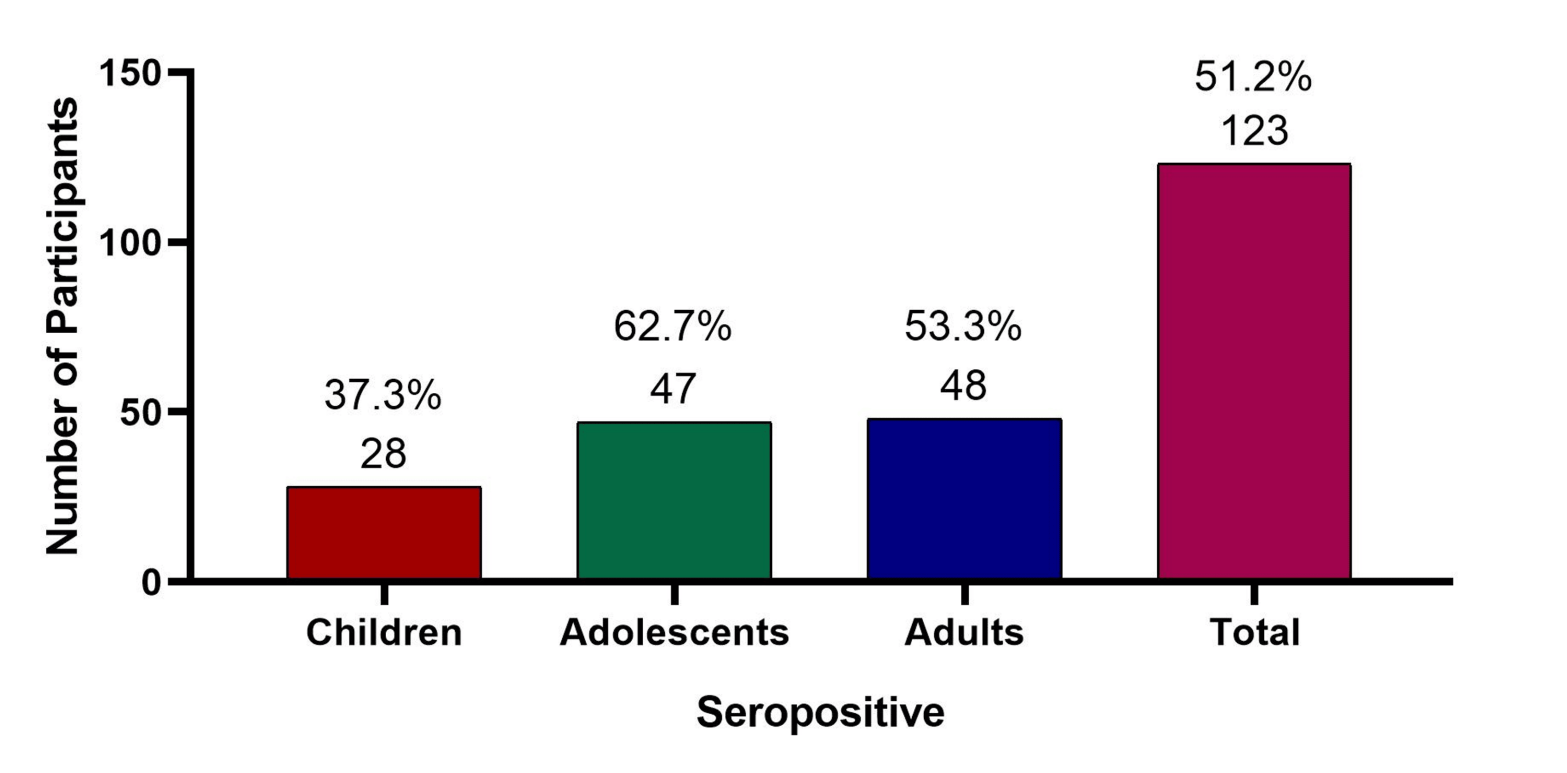
Seropositivity rates against dengue among different age groups.

Overall, dengue IgG seropositivity was observed in 123 participants, yielding a seroprevalence of 51.2% (95% CI: 44.9% to 57.5%). Age-wise seropositivity was 28/75 (37.3%, 95% CI: 26.4% to 48.2%) among children, 47/75 (62.7%, 95% CI: 51.8% to 73.6%) among adolescents, and 48/90 (53.3%, 95% CI: 43.0% to 63.6%) among adults (Figure 2).

**Figure 3:**
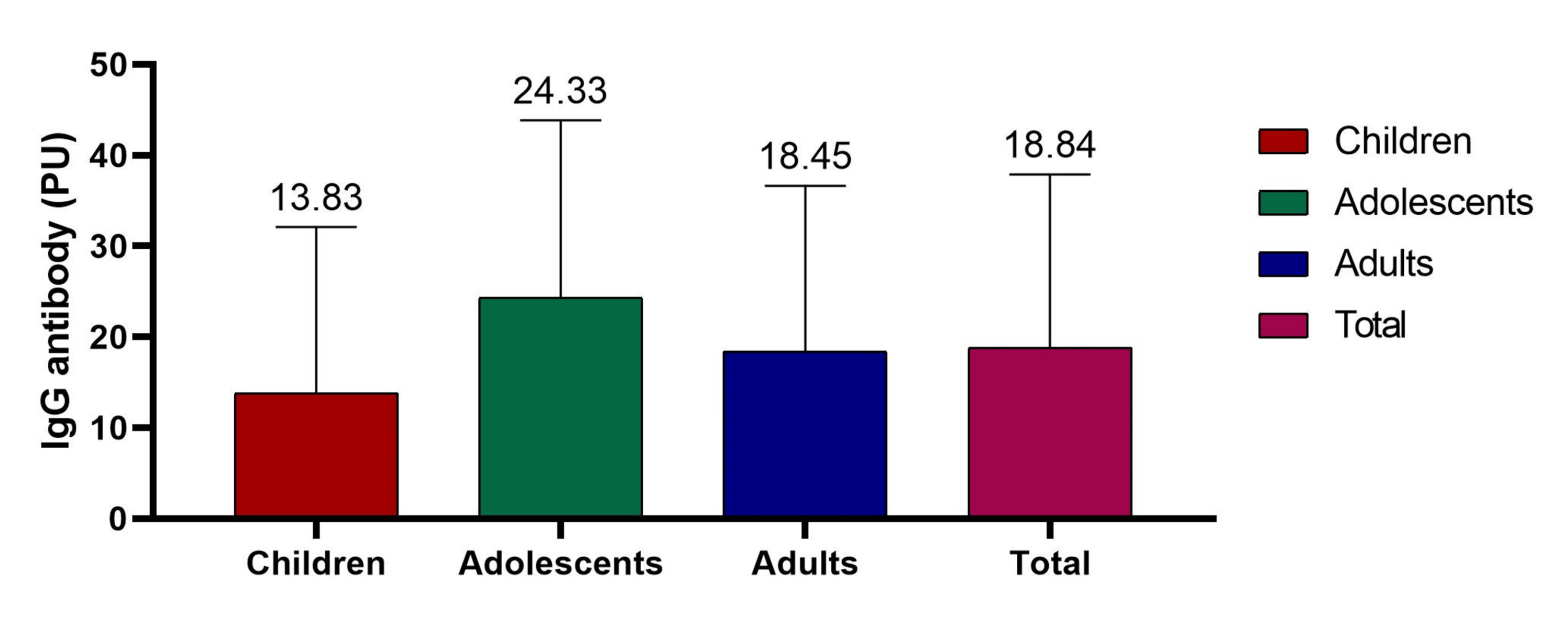
IgG antibody titers against dengue among different age groups (ANOVA test).

The mean IgG antibody titer (PU) among all participants was 18.84 ± 19.03. Age-wise mean IgG levels were 13.83 ± 18.26 in children, 24.33 ± 19.52 in adolescents, and 18.44 ± 18.21 in adults. One-way ANOVA demonstrated a statistically significant difference in mean IgG titers among the three age groups (F = 5.98, p = 0.003), with adolescents showing higher antibody levels compared to children and adults (Figure 3).

In bivariate analysis using the chi-square test, age group (χ² = 9.88, p = 0.007) and residence (χ² = 8.23, p = 0.004) were significantly associated with dengue IgG seropositivity. Urban participants had a higher seroprevalence (69/113; 61.1%) compared to rural participants (54/127; 42.5%). No significant association was observed with gender (χ² = 0.08, p = 0.773) or socioeconomic class (χ² = 1.10, p = 0.578) (Table 2).

**Table 2:** Bivariate association between sociodemographic variables and dengue IgG seropositivity.

Variable	Category	Positive n/N (%)	χ²	p-value
Gender	Male	65/129 (50.4%)	0.08	0.773
	Female	58/111 (52.3%)		
Age group	Children	28/75 (37.3%)	9.88	0.007
	Adolescents	47/75 (62.7%)		
	Adults	48/90 (53.3%)		
Residence	Urban	69/113 (61.1%)	8.23	0.004
	Rural	54/127 (42.5%)		
Socioeconomic class	Lower	45/86 (52.3%)	1.10	0.578
	Middle/Upper	78/154 (50.6%)		

In multivariable logistic regression analysis adjusting for age group and residence, gender was not independently associated with seropositivity (adjusted OR: 1.28; 95% CI: 0.73 to 2.24; p = 0.387). Age group demonstrated a significant overall association (Wald χ² = 9.81, p = 0.007). Compared with adults (25 to 30 years), children (nine to 12 years) had significantly lower odds of seropositivity (adjusted OR: 0.45; 95% CI: 0.23 to 0.87; p = 0.018), whereas adolescents did not differ significantly (adjusted OR: 1.31; 95% CI: 0.69 to 2.49; p = 0.406). Urban residence remained independently associated with higher odds of seropositivity compared to rural residence (adjusted OR: 2.16; 95% CI: 1.26 to 3.69; p = 0.005).

## Discussion

The WHO initially recommended conducting serosurveys to generate nationally representative seroprevalence data to inform decisions regarding the introduction of dengue vaccines in India [8]. According to nationwide survey results, approximately 49% of the population had evidence of prior DENV infection, although substantial regional variation was observed. Seroprevalence was higher in the northern, western, and southern regions compared to the northeastern and eastern regions [8]. The findings of the present study may similarly help identify age groups and geographic settings that could be considered in future vaccination and public health strategies.

The overall seropositivity rate in the present study was 51.2%, which is comparable to the North Indian study by Dinkar and Singh reporting a seropositivity of 51.22% [13]. In contrast, Ukey et al. from central India reported a lower overall seroprevalence of 31.3% [14]. Murhekar et al., in a population-based nationwide serosurvey, reported an overall seroprevalence of 48.7%, with age-specific rates of 28.3% among children aged five to eight years, 41.0% among those aged nine to 17 years, and 56.2% among individuals aged 18 to 45 years [8]. Recently, Soni and Bhatnagar reported a seroprevalence of 11.23% in Rajasthan [15]. International studies have also shown variability, with seroprevalence reported as 28.1% in Bangkok [16] and pooled IgG seroprevalence of 33.3% in Ghana [17]. These variations in seroprevalence across regions likely reflect differences in transmission intensity, climatic conditions, vector density, and study methodologies, underscoring the importance of region-specific data.

Dengue infection affects all age groups in India. Younger individuals are frequently affected during outbreaks, and children under 15 years are reported to be more susceptible to severe forms such as dengue hemorrhagic fever [18]. In the present study, the highest seropositivity (62.7%) was observed among adolescents (15 to 18 years), followed by adults (53.3%) and children (37.3%), suggesting increasing cumulative exposure with age. Similar age trends have been reported by Dinkar and Singh, who found the highest seropositivity in the 20-to-30-year age group [13], and by Ukey et al., who reported higher infection rates among individuals aged 15 to 30 years [14]. A meta-analysis reported a pooled median age of 22 years among laboratory-confirmed dengue cases [6]. However, some outbreaks have reported higher case numbers among younger age groups [18-21]. The variability in age distribution across studies may be influenced by outbreak dynamics, circulating serotypes, and prior immunity within the population.

The association between gender and dengue infection remains inconclusive. In the present study, no significant association was observed between gender and seropositivity in either bivariate or multivariable analysis. Several Indian outbreak studies have similarly reported male predominance [13,18,22,23]. Ukey et al. observed a male-to-female ratio of 2.15:1 among dengue cases [14]. However, Murhekar et al. reported no significant difference in seroprevalence between men (50.9%) and women (47.5%) [8], and some studies have reported higher infection rates among females [24,25]. Gender differences may reflect behavioral exposure patterns, occupational activities, healthcare-seeking behavior, or sociocultural factors rather than inherent biological susceptibility.

Dengue has traditionally been regarded as an urban disease, with most outbreaks initially reported from major Indian cities [20,26,27]. Urban and semi-urban areas with high population density and inadequate water management systems provide favorable breeding conditions for Aedes mosquitoes [9]. Rapid urbanization, industrialization, and environmental changes have further facilitated vector proliferation. In the present study, urban residence was significantly associated with higher seropositivity in both bivariate and multivariable analyses, and remained independently associated after adjustment. Similar urban predominance has been reported by Dinkar and Singh [13] and Murhekar et al. [8]. However, several recent studies have documented increasing seroprevalence in rural areas [6,18], and Ukey et al. reported a higher proportion of dengue-positive cases from rural settings [14]. These findings suggest that dengue transmission is no longer confined to urban centers and may be expanding into rural regions.

The present study has several strengths, including a robust sample size exceeding the calculated minimum and age-stratified analysis within the same population. However, as a hospital-based single-center study, selection bias cannot be excluded, and findings may not fully represent the general community. Participants attending outpatient services may differ from the broader population in healthcare-seeking behavior. Additionally, IgG seropositivity reflects prior exposure but does not distinguish between recent and remote infection, nor does it provide serotype-specific or neutralizing antibody information. The cross-sectional design precludes assessment of temporal trends or incidence.

## Conclusions

The present study demonstrated a high overall dengue IgG seroprevalence (51.2%) among individuals aged nine to 30 years in a North Indian tertiary care setting, indicating substantial cumulative exposure to the dengue virus. Seroprevalence was highest among adolescents, and children had significantly lower adjusted odds of seropositivity compared to adults. Urban residence was independently associated with higher seropositivity, suggesting intensified transmission in urban settings. These conclusions are supported by the statistically significant associations observed in multivariable analysis. No independent association was observed with gender or socioeconomic status. These findings highlight ongoing dengue transmission across age groups and underscore the importance of sustained vector surveillance, targeted public health interventions, and age-stratified monitoring in endemic regions. Seroprevalence data may contribute to informing context-specific vaccination considerations and control strategies, although population-based studies are required before policy decisions.
